# Polyploid plants take cytonuclear perturbations in stride

**DOI:** 10.1093/plcell/koae021

**Published:** 2024-01-24

**Authors:** Daniel B Sloan, Justin L Conover, Corrinne E Grover, Jonathan F Wendel, Joel Sharbrough

**Affiliations:** Department of Biology, Colorado State University, Fort Collins, CO, USA; Department of Ecology and Evolutionary Biology, University of Arizona, Tucson, AZ, USA; Department of Molecular and Cellular Biology, University of Arizona, Tucson, AZ, USA; Department of Ecology, Evolution, and Organismal Biology, Iowa State University, Ames, IA, USA; Department of Ecology, Evolution, and Organismal Biology, Iowa State University, Ames, IA, USA; Department of Biology, New Mexico Institute of Mining and Technology, Socorro, NM, USA

## Abstract

Hybridization in plants is often accompanied by nuclear genome doubling (allopolyploidy), which has been hypothesized to perturb interactions between nuclear and organellar (mitochondrial and plastid) genomes by creating imbalances in the relative copy number of these genomes and producing genetic incompatibilities between maternally derived organellar genomes and the half of the allopolyploid nuclear genome from the paternal progenitor. Several evolutionary responses have been predicted to ameliorate these effects, including selection for changes in protein sequences that restore cytonuclear interactions; biased gene retention/expression/conversion favoring maternal nuclear gene copies; and fine-tuning of relative cytonuclear genome copy numbers and expression levels. Numerous recent studies, however, have found that evolutionary responses are inconsistent and rarely scale to genome-wide generalities. The apparent robustness of plant cytonuclear interactions to allopolyploidy may reflect features that are general to allopolyploids such as the lack of F2 hybrid breakdown under disomic inheritance, and others that are more plant-specific, including slow sequence divergence in organellar genomes and preexisting regulatory responses to changes in cell size and endopolyploidy during development. Thus, cytonuclear interactions may only rarely act as the main barrier to establishment of allopolyploid lineages, perhaps helping to explain why allopolyploidy is so pervasive in plant evolution.

## Teaser

Despite predicted disruptions to cytonuclear interactions, recent studies suggest that (allo)polyploid plants are relatively robust to cytonuclear incompatibilities and stoichiometric imbalances.

## Predicted consequences of polyploidy for cytonuclear interactions

Polyploidy is one of the most widespread and consequential processes in plant genome evolution (Stebbins 1940; Wendel 2015; Van de Peer et al. 2021). Increases in nuclear genome copy number can occur within species (autopolyploidy) or in conjunction with hybridization between two species (allopolyploidy), although this dichotomy obscures a broad spectrum of genomic and taxonomic divergence between the parents involved in polyploid formation (Doyle and Sherman-Broyles 2017). Most studies of polyploidy in plants focus exclusively on genetic effects for the nuclear genome, without considering potential consequences for other genomic compartments, specifically the mitochondria and plastids. These cytoplasmic organelles evolved via endosymbiosis and still contain reduced versions of their ancestral bacterial genomes, encoding dozens of proteins, ribosomal RNAs (rRNAs), and transfer RNAs (tRNAs) (Timmis et al. 2004; Roger et al. 2017). Importantly, mitochondrial and plastid functions depend on coordinated interactions between these gene products and thousands of proteins that are encoded by nuclear genes and imported into the organelles from the cytosol (van Wijk and Baginsky 2011; Fuchs et al. 2020), many of which are the result of endosymbiotic gene transfer in early stages of eukaryotic evolution (Timmis et al. 2004; Gray 2015; Sloan et al. 2018). Therefore, changes in nuclear ploidy have the potential to affect these cytonuclear interactions, particularly when accompanied by hybridization. In this perspective, we argue that cytonuclear interactions may only rarely act as the main barrier to the establishment of allopolyploid lineages, perhaps helping to explain why allopolyploidy is so pervasive in plant evolution. A glossary of terms herein used is presented in Table 1.

**Table 1.** Glossary of terms

Term	Definition
Alloplasmic	The nuclear genome of one species or line paired with the organellar genomes of another species or line
Allopolyploid	A polyploid generated from two or more different genotypes, typically different species, but also applied to differentiated subspecies of a single species
Autopolyploid	A polyploid generated by doubling the chromosomes of a single genotype or species
Cytoplasmic male sterility	Male sterility conditioned by genes in organellar (usually mitochondrial) genomes
Diploidization	The evolutionary process of genome reduction following whole genome doubling, or polyploidy
Disomic inheritance	Genetic segregation patterns expected in a normal diploid organism with meiotic pairing between homologous (but not homoeologous) chromosomes
Endopolyploidy/Endoreduplication	The outcome of increased nuclear genome copy number in a cell resulting from DNA replication without subsequent cell division
F2 hybrid breakdown	Phenomenon where F2 and subsequent generations from wide crosses (e.g. between species) exhibit genotypes leading to defective or deleterious morphologies, even though the F1 appears normal or even heterotic
Fractionation	The process following polyploid formation in which loss of homoeologous genes results in a return to diploid copy number
Gene balance (as it applies to polyploidy)	Conceptual framework for understanding differential retention and loss of duplicated genes due to a pressure for stoichiometric expression with interacting partners who may also have been lost or preserved
Gene conversion	Recombination-mediated strand invasion and copy-correction of homologous pieces of DNA on different chromosomes—either alleles, paralogs, or in the special case of polyploidy, homoeologs
Homoeolog	Duplicated homologous copies of genes or chromosomes that result from polyploidy
Homoeologous exchange	Crossover products from recombination between homoeologous chromosomes
Homoploid hybrid	Interspecific hybrid having the same ploidy level as its parent species
N-mt gene	Nuclear gene encoding a protein that is targeted to the mitochondria
N-pt gene	Nuclear gene encoding a protein that is targeted to the plastids
Organellar (cytoplasmic) genome	An extranuclear genome, usually referring to DNA in mitochondria or plastids
Retrograde signaling	Signaling pathway from the mitochondria or plastids that regulates gene expression in the nucleus
Stoichiometry	Quantitative relationships among components of a system, e.g. polypeptides in protein complexes, ratios of organellar genome copy number to nuclear gene copy number, and ratios of nuclear volume to cell volume
Transit peptide	A short amino acid sequence at the N-terminus of some nuclear-encoded proteins that mediates import into the mitochondria and/or plastids and is cleaved during as part of the import process

**Figure 1.**
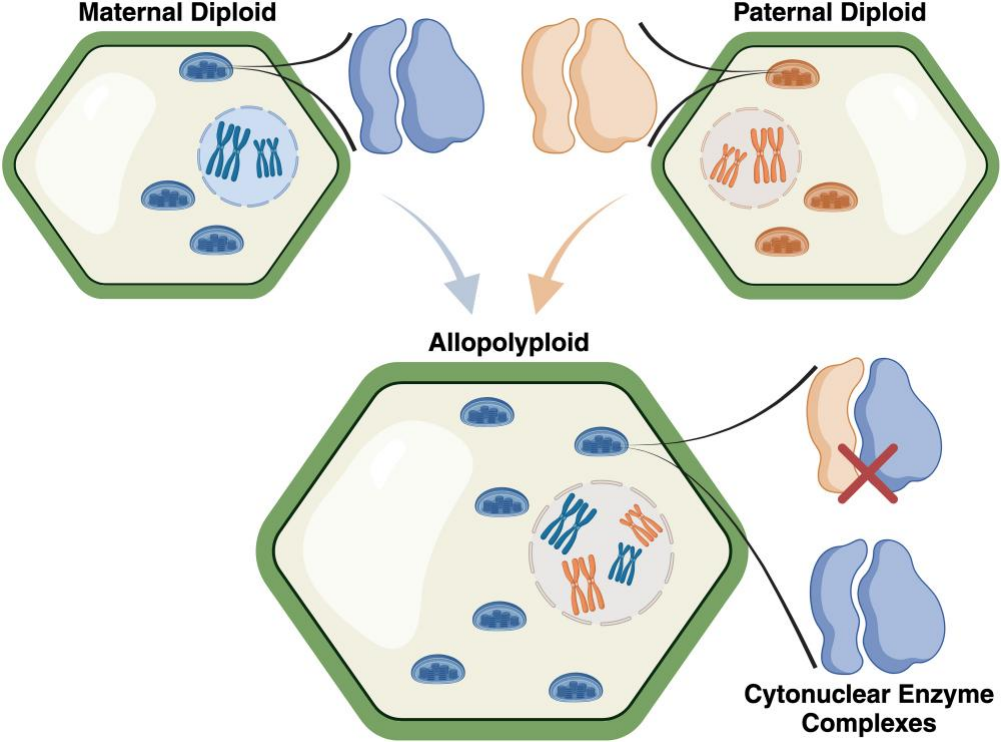
Allopolyploidy involves hybridization between two lineages and an associated doubling of the nuclear genome. The increase in genome size results in concomitant increases in nucleus size, cell size, and number of mitochondria (not pictured) and plastids. Whereas both parent species contribute a nuclear subgenome to the resulting allopolyploid lineage, the maternal progenitor is typically the sole source of the organellar genomes. Therefore, proteins encoded in the paternal nuclear subgenome and targeted to the mitochondria and/or plastids must interact with gene products from an evolutionarily novel and possibly divergent cytoplasmic background, potentially resulting in incompatibilities (as indicated by the red X). This figure was created with BioRender.com.

Two specific features of polyploidy have been hypothesized to perturb cytonuclear interactions (Sharbrough et al. 2017) (Table 2). The first is potentially relevant to both autopolyploids and allopolyploids and involves disruption of cytonuclear “gene balance” (Doyle and Coate 2019; Song et al. 2020; Birchler and Veitia 2021). Many molecular interactions occur in specific stoichiometric relationships. For example, the Rubisco enzyme complex is assembled from an equimolar ratio of plastid-encoded large subunits (RbcL) and nuclear-encoded small subunits (RbcS) (Andersson and Backlund 2008). All else equal, a doubling of the nuclear genome would be expected to produce an excess of nuclear-encoded subunits because of the 2-fold increase in nuclear gene copies. Studies of aneuploidy have indicated that nuclear–nuclear interactions can be highly sensitive to such imbalances (Birchler and Veitia 2012), but the stoichiometric effects of polyploidy on cytonuclear interactions have been less clear. The second hypothesized feature is specific to allopolyploids and relates to the contrasting transmission modes of nuclear vs. organellar genomes. Mitochondrial and plastid genomes are typically inherited from the maternal parent in a cross, although there are well-known exceptions to the rule of strict maternal transmission (Hagemann 2004; Gonçalves et al. 2020). Therefore, even in cases where allopolyploid populations are initially polymorphic for mitochondrial and plastid haplotypes due to reciprocal hybridization (Wendel and Doyle 2005), one cytoplasmic background is expected to eventually reach fixation within the lineage. For simplicity, we will refer to the donor of this cytoplasmic background as the maternal parent species. By contrast, both parent species contribute a full set of nuclear chromosomes to the hybrid lineage, resulting in maternally and paternally derived subgenomes. Therefore, the newly co-resident paternal nuclear subgenome and maternally inherited cytoplasmic background in allopolyploids have the potential to interact, despite having evolved in isolated lineages, sometimes for millions of years since last residing in a shared common ancestor (Sharbrough et al. 2022). Such cytonuclear “mismatches” have the potential to produce genetic incompatibilities and act as barriers to hybridization (Fig. 1) (Burton and Barreto 2012; Postel and Touzet 2020; Nakamura 2023).

**Table 2.** Perturbations to cytonuclear interactions in polyploids and hypothesized evolutionary responses

**Disruption of cytonuclear gene balance through doubling of nuclear gene copies**
*Evolutionary Responses:* Increase in cell size and number of mitochondria and plastids per cell
Increase in organellar genome copy numbers (per organelle)
Increase in expression level of organellar genes (per gene copy) Silencing/loss of nuclear genes that encode proteins that function in the mitochondria and plastids
**Incompatibilities between paternal nuclear subgenome and maternally inherited organellar genomes**
*Evolutionary Responses:* Replacement of paternal nuclear genes with their maternal counterparts via gene conversion and/or homoeologous exchange
Positive selection of amino acid substitutions in paternal proteins that restore compatibility
Preferential expression of maternal homoeologs for genes involved in cytonuclear interactions
Preferential retention of maternal homoeologs and loss of paternal homoeologs
Preferential import/assembly of maternal proteins

Several evolutionary responses have been predicted to offset these potentially disruptive effects of polyploidy on cytonuclear interactions (Table 2). For example, stoichiometric imbalances resulting from doubling of the nuclear genome could selectively favor increases in some combination of: (1) the number of mitochondria/plastids per cell; (2) the number of organellar genome copies per organelle; and (3) the level of gene expression per mitochondrial/plastid genome copy such that cytonuclear gene balance is restored. Likewise, cytonuclear incompatibilities in allopolyploids could result in compensatory processes that place asymmetric evolutionary pressures on the maternal vs. paternal nuclear subgenomes (Table 2). Paternally derived homoeologs (i.e. related nuclear gene copies originating from the two allopolyploid progenitors) could potentially experience positive selection for changes in amino acid sequence that restore compatibility with the mitochondrial and plastid gene products generated from the “novel” cytoplasmic background. Alternatively, selection may simply favor maternal homoeologs with mitochondrial and plastid functions, leading to the downregulation or loss of the corresponding paternal homoeologs. Such selection could be manifested in many ways, including preferential gene loss of paternal homoeologs, replacement of paternal homoeologs with maternal homoeologs via gene conversion or homoeologous exchange, gene expression bias towards maternal homoeologs and maternal expression levels, or discrimination against the proteins encoded by paternal homoeologs in the import/assembly process (see Sharbrough et al. 2017 and references therein). As discussed below, it is also possible that some of these responses could occur as a result of developmental or regulatory mechanisms and not require longer-term evolutionary changes mediated by selection.

Initial studies of individual species and genes provided helpful glimpses into whether these hypothesized cytonuclear perturbations and responses play an important role in the establishment of successful polyploid lineages (Bingham 1968; Bowman 1986; Gong et al. 2012; Oberprieler et al. 2019). In recent years, however, such investigations have expanded greatly both in taxonomic scope and methodological richness, testing species from a diverse sampling of angiosperm lineages at genome-wide scales. Therefore, it is an opportune time to consider this recent body of work and assess the generalities that may have emerged.

## Cytonuclear genome balance is immediately stabilized in polyploids

The notion that the doubling of the nuclear genome in polyploids will also produce a simple doubling of the ratio of nuclear to organellar genome copies appears to be an unjustified assumption. It has long been clear that nuclear polyploidy induces other changes such as increases in cell size and the number of organelles per cell (Doyle and Coate 2019). Indeed, earlier studies established that counting chloroplasts provided a reliable proxy for the ploidy level of a plant (Rhoades and Dempsey 1966; Bingham 1968; Butterfass 1987). Such scaling relationships between the number (or total volume) of organelles and genome/cell size are also evident in other eukaryotic lineages (Marshall 2020; Adams et al. 2024). In addition, there was early evidence that organellar genome copy number scaled with nuclear ploidy and genome size (Whiteway and Lee 1977; Bowman 1986; Shay et al. 1990). Expanded investigations have confirmed that elevated and approximately stoichiometric changes in organellar genome copy numbers per cell are a common feature across diverse angiosperm polyploid lineages (Coate et al. 2020; Fernandes Gyorfy et al. 2021). As such, cytonuclear genomic stoichiometry is largely stable across related species that differ in nuclear ploidy. It is not surprising, therefore, that cytonuclear stoichiometry of gene expression is also relatively stable across different nuclear ploidies—at least when measured as RNA transcript abundance and under benign conditions (Coate et al. 2020; Forsythe et al. 2022). Although the stoichiometry of cytonuclear interactions may not always maintain perfect proportionality across ploidy levels (Oberprieler et al. 2019), a clear picture has emerged that the scaling of organellar gene expression with nuclear ploidy often buffers polyploids against perturbations to genome balance and that this scaling starts at the level of increased numbers of organelles per cell.

The foregoing observations raise fundamental questions about the mechanisms and timing involved in stabilizing cytonuclear stoichiometry in polyploids. Does this stabilization depend on long-term evolutionary responses that require selection acting across generations to alter organellar genome copy number? Or is it an immediate developmental response to changes in nuclear ploidy? Evidence from studies of lab-generated polyploids increasingly supports the latter of these two possibilities. When polyploids are synthesized in the lab, they immediately exhibit the increased organellar genome copy numbers per cell and proportionality in cytonuclear gene expression that are also observed in more ancient polyploid lineages (Singsit and Ozias-Akins 1992; Ewald et al. 2009; Coate et al. 2020; Fernandes Gyorfy et al. 2021). Therefore, plants appear to have immediate cytoplasmic responses that compensate for changes in nuclear ploidy. These mechanisms may reflect existing regulatory pathways that are sensitive to changes in cell size and corresponding effects on surface area to volume ratios, as organelle shape and number are known to be environmentally and developmentally responsive (Jensen et al. 2000; Scott and Logan 2011; Greiner et al. 2020; Choi et al. 2021). Some responses may even just be a byproduct of basic cellular growth rates (Marshall 2020).

Whether this “immediate” response in stoichiometric balance that accompanies polyploidy also accompanies the well-known and much slower processes of diploidization following polyploidy remains a largely open question, as does the nature of the regulatory controls that operate in each direction. One long-term evolutionary response to tune cytonuclear stoichiometry in polyploids could act at the individual gene level by favoring the preferential retention or loss of duplicate nuclear genes encoding proteins that are targeted to the mitochondria (N-mt genes) or plastids (N-pt genes). Multiple studies have detected biased retention patterns for these gene classes in polyploid plants (Coate et al. 2011; De Smet et al. 2013; Li et al. 2016; Tasdighian et al. 2017; Emery et al. 2018; Ferreira de Carvalho et al. 2019; Sharbrough et al. 2022) and other eukaryotes (Wapinski et al. 2007; Conant 2014; Session et al. 2016; Li et al. 2018; Dimos and Phelps 2023). These biases, however, have been in different directions depending on the specific choice of loci, taxa, and temporal scale of analysis. For example, *Arabidopsis* N-pt genes exhibited preferential return to single-copy status following an ancient polyploidy event, while some N-mt genes involved in oxidative phosphorylation exhibited preferential retention (Emery et al. 2018). One theme that is potentially emerging from this body of work is that N-mt and N-pt genes are preferentially retained in young polyploids but that more ancient polyploids have experienced selection for these genes to preferentially return to single-copy status (Li et al. 2016). Therefore, the lengthy process of genome fractionation that follows polyploid formation might be one of the more significant sources of selection and perturbation to cytonuclear interactions. The dramatic increase in the number of identified polyploidy events in plant evolution and the accompanying availability of genome sequences present the opportunity to further test for this pattern. One hypothesis is that the larger cell sizes and lower surface area-to-volume ratios that result from genome doubling affect gas exchange and the efficiency of photosynthesis, creating selection pressure for subsequent diploidization (Wang et al. 2021). Such selection pressures may also be reflected in changes in cell morphology and physiology observed in polyploids (Domínguez-Delgado et al. 2021; Wilson et al. 2021).

Overall, it appears that longer-term responses related to cytonuclear stoichiometry in polyploids are complex and heterogeneous. Therefore, current evidence suggests that the immediate developmental responses to polyploidy are more consistent and predictable than the subsequent evolutionary fine-tuning of cytonuclear stoichiometry during diploidization.

## Inconsistent genome-wide signatures of selection against cytonuclear incompatibilities in allopolyploids

The hybrid nature of allopolyploids makes them potentially susceptible to cytonuclear incompatibilities (Burton and Barreto 2012; Postel and Touzet 2020; Nakamura 2023) between the paternal nuclear subgenome and the organellar genomes, which are typically inherited from the maternal progenitor (Camus et al. 2022). Early work indicated that there may be selection against such incompatibilities based on an apparent bias to replace or “overwrite” paternal homoeologs with sequence from maternal copies (Gong et al. 2012, 2014). This replacement process can be mediated by recombinational mechanisms, including strand invasions that result in unidirectional transfer of genetic material between related donor and recipient sequences (gene conversion) and reciprocal crossover events between homoeologous chromosomes that move large stretches of DNA (homoeologous exchange) (Gaeta et al. 2007; Gong et al. 2012; Mason and Wendel 2020; Lorenz and Mpaulo 2022). The early work on gene conversion in allopolyploids was based on a single cytonuclear enzyme complex (Rubisco), and subsequent studies have found that maternal bias is not always observed, at least not on shorter timescales (Sehrish et al. 2015; Wang et al. 2017; Ferreira de Carvalho et al. 2019; Zhai et al. 2019). Moreover, this replacement bias is only one of many potential mechanisms that have been hypothesized to alleviate cytonuclear incompatibilities in polyploids (Table 2). Accordingly, there have been recent efforts to test these a priori hypotheses in a genome-wide fashion across multiple allopolyploid lineages. The results have been decidedly mixed.

A recent analysis of rice (*Oryza*) allopolyploids reported that cytoplasmic background was associated with biased inheritance of specific regions of the nuclear genome that had been subject to homoeologous exchanges (Wu et al. 2021). However, these biases were at least as likely to favor the paternal genome copy as the maternal genome copy, indicating that the overall inheritance patterns do not appear to be driven by the “mismatch” between the cytoplasm and the paternal subgenome resulting from hybridization. In coffee (*Coffea arabica*), the predicted replacement bias in favor of maternal N-pt genes was observed in Rubisco, the plastid protease Clp, and the plastid NADH dehydrogenase-like (NDH) complex, but N-mt genes appeared to be especially resistant to homoeologous recombination (in either direction) (Ortiz and Sharbrough 2023). In cotton (*Gossypium*) allopolyploids, no maternally biased replacement was observed for either N-pt or N-mt genes (Conover et al. 2023). In fact, detailed analysis to distinguish true gene conversion from other mechanisms that can produce similar patterns of sequence variation suggested that conventional “4-taxon” phylogenetic approaches may have high false positive rates when inferring gene conversion events (Conover et al. 2023), and that previous findings using this approach should be interpreted with caution. Collectively, these studies have revealed scant evidence of cytonuclear selection favoring maternally biased gene replacement following allopolyploidy.

An alternative mechanism that could mitigate cytonuclear incompatibilities is positive selection for amino acid substitutions in paternal N-mt and N-pt proteins that restore functional interactions. Few if any studies have demonstrated this phenomenon empirically, but some data hint at the possibility. For example, Gong et al. (2012) reported that the two duplicated copies in five allopolyploid cotton species encoding RbcS are identical to each other and to the maternal homolog but different from the paternal diploid copy at a specific amino acid residue. There is also the possibility that a reduced functional role of paternal homoeologs would relax purifying selection on these genes. Both positive selection and relaxed purifying selection would be expected to lead to faster rates of evolution in paternal homoeologs than in their maternal counterparts. However, a recent genome-wide analysis across six independently formed angiosperm allopolyploid lineages did not detect any such bias in rates of protein sequence evolution (Sharbrough et al. 2022).

Rather than changing the sequence of paternal N-mt and N-pt proteins in allopolyploids via homoeolog replacement or de novo substitutions, an alternative mechanism that could alleviate cytonuclear incompatibilities is downregulation of these paternal genes in favor of expression of their maternal homoeologs. Numerous recent studies have tested this prediction through RNA-seq analysis (Ferreira de Carvalho et al. 2019; Shan et al. 2020; Bird et al. 2021; Burns et al. 2021; Li et al. 2021; Grover et al. 2022). It is well established that the two subgenomes in allopolyploids can contribute differentially to gene expression (Alger and Edger 2020; Bird et al. 2021); however, there has been limited support for the prediction that these biases preferentially affect N-mt and N-pt genes or consistently favor the maternal subgenome. For example, recently formed *Tragopogon* allopolyploids did not show evidence of cytonuclear effects on expression bias (Shan et al. 2020). A broader survey of six angiosperm allopolyploid lineages that span a large range of different ages revealed that the magnitude and direction of subgenome bias was highly variable across species and among different functional categories (Grover et al. 2022). Analysis of the allopolyploid *Arabidopsis suecica* found that homoeologs exhibiting biased expression were enriched for N-pt genes, but this bias favored paternal expression (Burns et al. 2021). The authors proposed that this bias could reflect an adaptive response by paternal genes that have to function in a novel cytoplasmic background, but this post hoc interpretation is opposite the evolutionary response to cytonuclear incompatibilities that has typically been predicted. Multiple studies have investigated duplicate gene expression in *Brassica* allopolyploids (Ferreira de Carvalho et al. 2019; Bird et al. 2021; Li et al. 2021). These studies have shown evidence of maternal bias and preferential effects on genes with cytonuclear function in resynthesized allopolyploids, but these effects appear to be attenuated or undetectable in the established allopolyploids. In sum, this collection of studies has pointed to highly species-specific and gene-specific patterns of cytonuclear expression bias rather than responses that consistently favor maternally biased expression of N-mt and N-pt genes at a genome-wide level.

The most extreme form of downregulating a gene is to lose it entirely from the genome. Therefore, another possible mechanism to favor maternal N-mt and N-pt genes is their preferential retention, while paternal homoeologs are lost in the wake of allopolyploid formation. Analysis of five independent angiosperm allopolyploid lineages found that subgenomes tend to differ significantly in total gene content (Sharbrough et al. 2022). When adjusting for these subgenome-wide patterns, *Brachypodium hybridum* exhibited preferential retention of maternal N-mt and N-pt genes, as expected if gene retention patterns reflect selection to preserve coevolved cytonuclear interactions. However, *Chenopodium quinoa* and *Triticum dicoccoides* exhibited the opposite pattern with a paternal bias (and two other species exhibited no significant bias either way when controlling for overall differences between subgenomes). Therefore, as with expression level biases, there does not appear to be a consistent pattern across lineages for preferential retention of maternal N-mt and N-pt genes.

In contrast to the extensive work on gene expression at the level of mRNA transcript abundance, much less has been done to investigate biases in protein translation, import, assembly, and turnover, all of which have the potential to influence the relative functional contributions of maternal and paternal homoeologs to cytonuclear interactions (Soltis et al. 2016). These translational and post-translational processes involve machinery that is mostly or entirely nuclear-encoded. For example, nuclear-encoded proteins are translated by cytosolic ribosomes, which are entirely nuclear-encoded themselves. Likewise, with only a few exceptions (Kikuchi et al. 2013; Carrie et al. 2016; Kikuchi et al. 2018), the import machinery that translocates proteins into the mitochondria and plastids is also nuclear-encoded. Nonetheless, there are some hints that processes such as protein import and assembly might play a role in stabilizing cytonuclear interactions in allopolyploids. For example, allohexaploid wheat experienced a paternal-to-maternal gene conversion event in the region of a nuclear *RbcS* gene that encodes the transit peptide required for protein targeting and import into the plastids (Li et al. 2020). In addition, paternal-to-maternal gene conversions and biased expression of maternal homoeologs has been documented for some of the nuclear-encoded chaperones that function in assembly of Rubisco enzyme complexes (Li et al. 2022). Whether examples like this are driven by selection against cytonuclear incompatibilities remains unclear, but they point to a relatively unexplored area in the stabilization of allopolyploid cytonuclear interactions that merits further investigation.

## Why do not plant polyploids exhibit more genome-wide signatures of selection on cytonuclear interactions?

As summarized above, recent investigations have revealed surprisingly inconsistent signatures of selection on cytonuclear interactions in the wake of hybridization and whole genome duplications in plants. These findings suggest that plant cytonuclear interactions are more robust to the disruptive effects of allopolyploidy than previously posited. Some of this robustness might reflect general features that are common to all polyploids (i.e. not specific to plants). For example, one of the key ways in which genome doubling is likely to stabilize hybrid lineages is by preventing the process of F2 hybrid breakdown that occurs in homoploid (i.e. diploid) hybrids. This process involves segregating out homozygous genotypes from the two original progenitors, thereby exposing incompatibilities that may have been masked in the F1 heterozygous state. Accordingly, many cytonuclear incompatibilities in diploids are not revealed until the F2 generation (Burton and Barreto 2012). Allopolyploids, however, typically exhibit disomic inheritance (i.e. the meiotic pairing of homologous but not homoeologous chromosomes) such that chromosomes from each progenitor are consistently transmitted across generations and “heterozygous” masking effects persist (Otto 2007).

More specific features of plant biology may also contribute to the apparent robustness of plant cytonuclear interactions. For example, in contrast to many other eukaryotic lineages, the mitochondrial and plastid genomes of land plants are notable for their extremely low rates of gene sequence evolution (Wolfe et al. 1987). Recent modeling suggests that the generation of co-adapted cytonuclear interactions via compensatory coevolution may require high mutation rates in organellar genomes (Lynch 2023). Therefore, the slow mitochondrial and plastid mutation rates typical of plants may lead to retention of cytonuclear compatibility across larger timescales of divergence. This hypothesis is consistent with observations that some parasitic and host plants can undergo extensive horizontal gene transfer of mitochondrial and N-mt genes with little indication of positive selection to restore compatibility (Ceriotti et al. 2022). A handful of plant lineages (e.g. Geraniaceae, *Plantago*, and *Silene*) with atypically high rates of mitochondrial and plastid genome evolution may also provide the exception that proves the rule. These taxa exhibit much stronger genome-wide signatures of selection on cytonuclear interactions (Zhang et al. 2015; Havird et al. 2017; Forsythe et al. 2021) that are more akin to observations in animals (Barreto et al. 2018; Yan et al. 2019). Therefore, variation in rates of mitochondrial and plastid sequence evolution across lineages may be an important determinant of selection on N-mt and N-pt genes following polyploidy events (Zuo et al. 2022). Future investigations could also consider the gene-specific consequences of cases in which there is substantial rate variation across regions within organellar genomes (Magee et al. 2010; Zhu et al. 2014).

In contrast to the typically slow rates of point mutations and gene sequence evolution, the rate of structural rearrangements is very high in angiosperm mitochondrial genomes. These rearrangements can produce chimeric sequences that disrupt anther development and pollen production in a phenomenon known as cytoplasmic male sterility (CMS) (Horn et al. 2014). Nuclear restorer-of-fertility genes coevolve to silence these CMS elements (Fujii et al. 2011), but generation of mismatched nuclear and organellar genomes through hybridization can reveal male sterility phenotypes. Indeed, CMS is one of the most common incompatibilities found in angiosperm hybrids (Postel and Touzet 2020), supporting the idea that mutation rates in organellar genomes are a key determinant of the rate and type of cytonuclear incompatibilities that are observed.

In both allopolyploids and autopolyploids, immediate changes in cell size and organellar genome copy number may blunt potential disruptions to cytonuclear gene balance and associated selection pressures. Normal developmental processes in plants may have predisposed them to tolerate shifts in nuclear genome copy number. For example, the “alternation of generations” that is characteristic of plant reproductive cycles requires extended development periods functioning at different ploidy levels (diploid sporophytes and haploid gametophytes). In addition, development of vegetative tissues often involves rounds of DNA replications without cell division, resulting in large increases in nuclear ploidy (known as endoreduplication or endopolyploidy). Therefore, even more so than other eukaryotic lineages, plants may have evolved regulatory responses that coordinate mitochondrial and plastid function with changes in nuclear ploidy and cell size (Kawade et al. 2013; Pacey et al. 2020; He et al. 2021).

## Caveats: reasons why genome-wide investigations may not detect legitimate cytonuclear effects in polyploid formation

Despite the inconsistent results from recent studies, nuclear genome doubling clearly has effects on cytonuclear interactions, if only from first principles based on cellular and nuclear volumes and all of the cascading scaling issues that can influence gene expression (Doyle and Coate 2019; Marshall 2020; Sun et al. 2020). Therefore, it is worth asking whether current approaches may systematically underestimate cytonuclear responses to polyploidy or if they lack the statistical power to detect them. For example, the genome-wide nature of many recent studies may represent both a strength and a weakness. These studies have the advantage of comparing thousands of genes that are partitioned in an unbiased fashion according to a priori predictions (e.g. N-mt and N-pt genes vs. the rest of the genome), but at the risk of diluting any biological signal that is limited to a small number of nuclear genes involved in cytonuclear interactions (or even individual nucleotides or amino acid residues). Analyses based on genome-wide partitioning of N-mt or N-pt genes could also be misleading when other types of genes are targets of selection for cytonuclear compatibility. For example, the breeding history of alloplasmic wheat lines provides a clear example of cytonuclear incompatibilities contributing to the success or failure of establishing allopolyploid plants (Nakamura 2023). Notably, the only contributing nuclear locus that has been mapped to a single candidate gene in these wheat lines encodes a protein that does not appear to be localized to the mitochondria or plastids (Bassi et al. 2016). Instead, based on its sequence similarity to RHOMBOID-LIKE 2 in *Arabidopsis* and its lack of a predicted N-terminal targeting peptide, this protein potentially plays a role in mitochondrial retrograde signaling by cleaving transcription factors anchored to the endoplasmic reticulum (Eysholdt-Derzsó et al. 2023). It is easy to envision how such effects would not be detectable in analyses conducted within the framework of subcellular targeting.

Studies testing for evolutionary responses to deleterious cytonuclear effects in polyploids may also suffer from an obvious ascertainment or survivorship bias. Investigations of established polyploids are necessarily conducted on the “winners.” Polyploids that experience severe cytonuclear incompatibilities or dosage effects will likely fail before they can even become established, suggesting that selection in successful lines will only have had to act on weak cytonuclear effects. Therefore, continued use of natural polyploids that have formed very recently (Shan et al. 2020) or lab-generated “synthetic” polyploids (Coate et al. 2020) is important to mitigate this bias.

Another consideration is that most functional genomic analyses of gene expression in polyploids have been conducted on bulk tissue samples, potentially obscuring biologically meaningful patterns at the level of individual cells. A recent single-cell RNA-seq analysis of multiple angiosperm allopolyploids found that the extent of maternal or paternal expression bias for N-mt and N-pt genes varied substantially across cell types and over time (Zhang et al. 2023). These findings raise questions about whether there are mechanisms (and associated testable predictions) that would have been selected to preferentially maintain coadapted cytonuclear partners in certain cell types or developmental timepoints.

Finally, as noted above, nearly all studies investigating maternal vs. paternal bias in plant allopolyploids have focused on the genomic and/or transcriptomic levels. Therefore, protein-level mechanisms related to translation, import, assembly, and turnover remain a relatively untested arena for evolutionary responses to cytonuclear incompatibilities in allopolyploids.

## Conclusion: cytonuclear interactions—a leaky gatekeeper in plant allopolyploidy?

The recent and ongoing proliferation of genome-wide datasets in numerous polyploid plant systems has created opportunities to test long-standing hypotheses about the disruptive effects of nuclear genome doubling on interactions with organellar genomes. Surprisingly, many effects that were predicted and even supported by early studies of individual genes have not emerged as generalities at a genome-wide level. Perturbations to cytonuclear stoichiometry appear to be ameliorated by developmental, growth, and regulatory responses that stabilize at least some aspects of cytonuclear gene balance even upon initial formation of polyploids. In allopolyploids, evidence has been inconsistent regarding predicted biases against the paternal subgenome in patterns of gene loss, homoeolog replacement, sequence evolution, and gene expression. We conclude that cytonuclear incompatibilities from hybridization are insufficiently widespread or uniform to be reliably detected in genome-wide partitions based on simple features such as mitochondrial and plastid targeting or assembly within cytonuclear enzyme complexes. Many features of allopolyploids in general and plants in particular may make them robust to cytonuclear incompatibilities. Furthermore, when cytonuclear incompatibilities do occur in allopolyploids, the genetic basis may be idiosyncratic and taxon-specific. Therefore, after zooming out to test for “global” generalities across whole genomes and diverse taxa, this field appears poised to zoom back in to identify specific genetic interactions in individual taxa. Recent advances in areas such as organellar genome editing, structural biology of cytonuclear enzyme complexes, and cytonuclear signaling pathways provide an expanding toolkit for combining forward and reverse genetics to address these challenges.

## Dive Curated Terms

The following phenotypic, genotypic, and functional terms are of significance to the work described in this paper:

NADH CHEBI: CHEBI:16908

initial AmiGo: PO:0004011
